# E-Nephrology

**DOI:** 10.4103/0971-4065.78295

**Published:** 2011

**Authors:** P. K. Manchanda, H. K. Bid

**Affiliations:** Department of Endocrinology, Internal Medicine and James Cancer Center, The Ohio State University, Columbus, Ohio, USA; 1 Center for Childhood Cancer, The Research Institute at Nationwide Children’s Hospital, Columbus, Ohio, USA

**Keywords:** Nephrology, internet, web

## Abstract

Diagnosis of renal diseases is often delayed owing to the scarcity of trained physicians, lack of facilities, and shortage of funds limits effective management, particularly when it comes to the red zone of renal replacement therapy. The Internetis expected to open up a myriad resource of knowledge and applications for academicians, researchers and clinicians alike in all health care professions across the globe. Also, the Internet has grown rapidly over the years and will inevitably expand even more. Evolving technologies offer modern applications for information management, communications with multimedia and virtual reality. Now, these innovative technologies have opened up newer possibilities for nephrologists. As Internet is serving as a backbone for these modern technologies, it is an utmost necessity to use and refine Internet applications for future nephrologists. Increasingly easy access to Internet has dramatically reduced barriers in sharing of information among basic and clinical nephrologists. Considering the growing scope for nephrologists in the use of Internet, it is necessary to understand Internet as a source of information and backbone of modern application. This review illustrates expanding roles of the Internet for the nephrologists and provides ready to use compilation of useful academic, research, clinical resources and is expected to introduce, stimulate and guide nephrologists into the realm of the world wide web. It also investigates how Internet is supporting in growth and development of the field of nephrology and present and future scopes of Internet as a tool for professionals involved in this area as well as information about biological sciences, and it also gives information about societies in various continents working in field of nephrology and the links useful for clinicians and research scientists.

## Introduction

The Internet is set to be in the forefront of the new age of technological revolution. With the explosive growth of the World Wide Web in recent months, health and medical websites are evolving. The Internet is a colossus body of information, which has, in recent years, witnessed an amazing expansion to such an extent that colloquially it is now termed as the “information superhighway.” There has been an enormous increase in the amount of information available through the Internet over the recent years. With an estimated 320 million web pages available,[1] it has emerged as the most powerful platform for exchange of scientific knowledge.[2]

Nowadays, Internet has become one of the most common media to explore and extract information of interest for researchers in any field, including nephrology. Its power is most strongly seen among scientists, as more and more information is made available through “the Net,” whether it is gene sequences, experimental data, chromosome maps or complete journal articles.[3] Its influence is most strongly felt among basic scientists and clinicians as more and more information is coming on “the Net” in the form of e-journals, e-books, research articles, gene sequences, experimental data or chromosome maps.[4] In the past few years, many Internet and nephrology resources have been posted on the web, including medical journals, medical associations, clinical trial registries and medical guidelines. Solze *et al*[5], have discussed in their review about online texts and web resources that are, in many respects, ahead of other areas of medicine and websites which provide free full-text access to medical journals and books in poorer countries: the Global Health Network Supercourse, which provides specially designed online lectures for the developing world, and Internet2/Abilene and similar research networks around the world, which provide reliable, guaranteed bandwidth for high-quality Internet videoconferencing as an alternative to face-to-face lectures and meetings for nephrologists.

## Origins of the Internet

Internet originated as a defense computer network (ARPANET) initiated by ARPA of US Defense, later known as Defense Advanced Research Projects Agency (DARPA). This ARPANET grew into different phases of development open architecture format with modified protocols and finally developed into the present day Internet, creating the WWW as a platform for exchange of information between geographically separated computers. The Internet and WWW (also called W3 and the Web) have morphed into a sort of tool for retrieval and disposal of scientific and other literature for the advancement of education. It is Internet’s most exciting and popular development. As Internet matured into global treasure of ever growing knowledge, it touched all spheres of life. A list of few common websites has been recently reported by Konwar *et al* [Table 1].[6]

**Table 1:** List of important website addresses related to health care information resources for kidney diseases

Kidney diseases	Links
Kidney diseases from the U.S. NIDDK	http://www.niddk.nih.gov/health/kidney/kidney.htm
National Kidney Foundation from the U.S.	http://www.kidney.org/
American Association of Kidney Patients information, education, support	http://www.aakp.org/
RenalWorld kidney disease, dialysis and transplantation resources from around the world	http://renalworld.com/
National Kidney and Transplant Institute providing health and medical services in the Philippines	http://kidney.gov.ph/
Kidney Dialysis Foundation in Singapore, helping the needy	http://www.kdf.org.sg/
Life Options Rehabilitation Program for dialysis patients, sponsored by Amgen Inc.	http://www.lifeoptions.org/
National Home Hemodialysis Program operated by the Lower Manhattan Dialysis Center in NYC	http://www.homehemoonline.com/
The Kidney Stone WebSite an educational resource for those who suffer from kidney calculi	http://members.aol.com/rogerbaxtr/pages/Kidney_Stone_Page.html
Kidney Stones from the American Foundation for Urologic Disease	http://www.afud.org/conditions/ks.html
IgA Nephropathy Foundation Berger’s disease, the most common non-diabetic kidney disease	http://www.igan.org/
Kidney Stones from eMedicine’s consumer health collection	http://www.kidneystones.e-medicinehealth.com/
Polycystic Kidney Disease Information Page bulletin board, chat room, links and information	http://www.geocities.com/HotSprings/Spa/3265/
Adult Polycystic Kidney Disease info from the U.S. NCBI about the genetics of PKD	http://www.ncbi.nlm.nih.gov/disease/PKD.html
World Kidney Fund aiding kidney sufferers in developing countries	http://www.worldkidneyfund.org/
Kidney Patient Guide from the U.K., supported by the Wellcome Trust	http://www.kidneypatientguide.org.uk/
NephCure Foundation seeking a cure for Nephrotic Syndrome and Focal Segmental Glomerulosclerosis	http://www.nephcure.org/
Kidney and Urology Foundation of America avoiding the debilitating effects of kidney/urologic diseases	http://www.kidneyurology.org/
American Lithotripsy Society for physicians and others at lithotripsy sites in the U.S.	http://www.gamewood.net/rnet/renalpath/tutorial.htm
Renal World	http://renalworld.com/regions_links/index_html

### Searching the Web

Internet has grown-up into a colossal mass of information, and searching Internet for information requires skills to avoid wasting time and obtain maximum relevant biomedical information. Basically, Internet can be searched for information by using i) crawlers, ii) human-catalogued directories, iii) hybrid search engines, iv) specialized search tools, v) meta search tools and vi) tool bars. Now some specialized search engines are also coming up like Kartoos (http://www.kartoo.com/) which presents results in graphical ma format, while Vivisimo (http://vivisimo.com) presents results as clusters of related terms. Metasearch tools search through different search engines at one time and compare them to find out the best suitable results.[7] All the available search engines have their advantages and disadvantages and should be selected on the basis of specific search requirement. More about search engines can be learnt from websites dedicated to search engine (http://searchenginewatch.com).

### Really Simple Syndication

Really Simple Syndication (RSS) is an excellent technology that is being used by nephrologists around the world to keep track of their favorite websites. RSS flips things around a little and is a technology that provides you with a method of getting relevant and up to date information sent to you, for you to read in your own time. It not only saves time but also gets the information quickly after it is published.[8]

### Web 2.0

Web 2.0 resources impede creativity, collaboration and sharing among users. Integration of Web 2.0 resources allows scientists, students, and medical professionals/nephrologists to efficiently organize and manage the information and resources that are critical in today’s quickly changing biomedical research environment. The recognition of the term Web 2.0, along with the growing use of blogs, wikis and social networking technologies, has led many in academia and business to coin a outbreak of 2.0s, including Library 2.0, Social Work 2.0, Enterprise 2.0, PR 2.0, Classroom 2.0, Publishing 2.0, Medicine 2.0, Telco 2.0, Travel 2.0 and Government 2.0.

### eTOCs

The eTOC service allows anyone who registers their e-mail address to be notified via e-mail when new content goes online. You may choose to receive any or all of the following: notification that a new issue of health information on the Internet is online, complete table of contents for new issues and special announcements from nephrology journals. Virtually, all the key nephrology journals are now available electronically and offer an e-mailed table of contents (eTOC) service whereby nephrologists who register can be notified via e-mail when new content goes online.

## Nephrology and Internet

Internet has provided an interactive forum for the researchers and clinicians working in nephrology

A large and rapid growing volume of biomedical information can be accessed through the Internet. There are many databases available on Internet that may be very useful not only to the nephrologists but also to the people from other streams. At present, numerous biomedical databases of journals are accessible in Internet which include index of journal citations and abstracts of articles and even links to full text articles. Table 2 enlists few of the preferred sites for searching scientific literatures in nephrology. Some professional organizations related to nephrology are listed in Table 3 which helps not only the basic researcher but also clinical nephrologists.

**Table 2:** List of journals related to nephrology

Advances in Chronic Kidney Disease	http://journals.elsevierhealth.com/periodicals/xackd
Advances in Chronic Kidney Disease	http://www.us.elsevierhealth.com/product.jsp?isbn=15485595
Advances in Renal Replacement Therapy	http://www.us.elsevierhealth.com/product.jsp?isbn=10734449
American Journal of Hypertension	http://www.journals.elsevierhealth.com/periodicals/ajh
American Journal of Kidney Diseases	http://journals.elsevierhealth.com/periodicals/yajkd
American Journal of Kidney Diseases	http://www.ajkd.org/
American Journal of Physiology: Renal Physiology	http://ajprenal.physiology.org/
American Journal of Transplantation	http://www.medscape.com/viewpublication/1005_index
Atlas of diseases of the kidney	http://www.kidneyatlas.org/
BMC Nephrology	http://www.biomedcentral.com/bmcnephrol/
Clinical and Experimental Nephrology	http://link.springer-ny.com/link/service/journals/10157/index.htm
Current Opinion in Nephrology & Hypertension	http://www.co-nephrolhypertens.com
Dialysis Centre	http://www.globaldialysis.com/centres.asp
Hong Kong Journal of Nephrology	http://asia.elsevierhealth.com/hkjn/
Hypertension Dialysis Clinical Nephrology Electronic Journal	http://www.hdcn.com/
Hypertension	http://hyper.ahajournals.org/
Indian Journal of Nephrology	http://www.indianjnephrol.org
International urology and nephrology	http://www.springerlink.com
Jornal Brasileiro de Nefrologia	http://www.sbn.org.br/JBN/jbnsearch.htm
Journal of Clinical Hypertension	http://www.medscape.com/viewpublication/110_index
Journal of Renal Nutrition	http://www.us.elsevierhealth.com/product.jsp?isbn=10512276
Journal of the American Society of Nephrology	http://jasn.asnjournals.org/
Kidney & Blood Pressure Research	http://content.karger.com/
Kidney International	http://www.blackwellpublishing.com/journal.asp?ref=0085-2538&site=1
Kidney patient guide	http://www.kidneypatientguide.org.uk/
Medical News Nephrology	http://www.medicalnews.com/nephrology
National Kidney Foundation	www.kidney.org
Nephrology	http://www.blackwellpublishing.com/journal.asp?ref=1320-5358&site=1
Nephrology Dialysis Transplantation	http://ndt.oxfordjournals.org/
Nephrology nursing journal	http://nephrologynursing.net/
Nephron Clinical Practice	http://content.karger.com
Nephron Experimental Nephrology	http://content.karger.com/
Nephron Physiology	http://content.karger.com
Pediatric Nephrology	http://www.springerlink.com
Peritoneal Dialysis International	http://www.ispd.org/pdi_journal.html
Peritoneal Dialysis Today	http://www.multi-med.com/pdtoday/
Renal Failure	http://ns.gamewood.net/rnet/rf/rfhome.htm
Scandinavian Journal of Urology and Nephrology	http://www.tandf.co.uk/journals/titles/00365599.asp
Seminars in Nephrology	http://journals.elsevierhealth.com/periodicals/ysnep
The American Journal of Physiology - Renal Physiology	http://ajprenal.physiology.org/
The Electronic Kidney	http://www.kidney.org/eleckid.html
The Internet Journal of Nephrology	http://www.ispub.com/ostia/index.php?xmlFilePath=journals/ijne/front.xml
Transplant International	http://www.springerlink.com

**Table 3:** List of different organizations related to nephrology

Organizations/Agencies	Web address
International Society for Hemodialysis (ISHD)	http://www.ishd.net/about.php
American Kidney Fund	http://www.akfinc.org/
American Nephrology Nurses Association	http://www.annanurse.org/cgi-bin/WebObjects/ANNANurse
American Society of Diagnostic and Interventional Nephrology	http://www.asdin.org/
American Society of Nephrology : ASN	http://www.asn-online.org/
Arab Society Of Nephrology and Renal Transplantation	http://www.asnrt.org/
Asian Pacific Society of Nephrology	http://www.apsneph.org/
Association of Renal Technicians	http://www.artery.org.uk/
Australian and New Zealand Society of Nephrology	http://www.nephrology.edu.au/
Australian Kidney Foundation	http://www.kidney.org.au/
Brazilian Society of Nephrology	http://www.sbn.org.br/
Canadian Association of Nephrology Nurses and Technologists	http://www.cannt.ca/links
Canadian Society of Nephrology	http://rcpsc.medical.org/index.php?pass=1
Danish Society of Nephrology	http://www.dns.suite.dk/
Egyptian Society of Nephrology	http://163.121.19.91/esnnew/
European Renal / Dialysis and Transplant Association	http://www.era-edta.org/erafset.htm
European Renal Association	http://www.era-edta.org/erafset.htm
European Renal Association	http://www.era-edta-reg.org/index.jsp
European Society for Pediatric Nephrology	http://espn.uwcm.ac.uk/
Hellenic Society of Nephrology	http://www.ene.gr/
Hereditary Nephritis Foundation	http://www.cc.utah.edu/~cla6202/HNF.htm
Indian Society of Nephrology	http://www.isn-india.com/
International Society for Hemodialysis	http://www.ishd.net/
International Society of Nephrology	http://www.isn-online.org/site/cms/
Italian Society of Nephrology	http://www.sin-italia.org/
Japanese Society of Nephrology	http://www.jsn.or.jp/
Kidney Epidemiology and Cost Centre USRDS	http://www.med.umich.edu/usrds/
Kidney Foundation of Canada	http://www.kidney.ca/index-eng.html
Korean Society of Nephrology	http://www.ksn.or.kr/
Libyan Society Of Nephrology	http://www.nephrolibya.org/
Malaysian Society of Nephrology	http://www.msn.org.my/index.jsp
National Kidney Foundation	http://www.kidney.org/
National Kidney Foundation Singapore	http://www.nkfs.org/main.htm
Nephrologists Forum	http://www.he.net/~brumley/renal/doctorboard.html
Renal Pathology - Web Path, University of Utah	http://www.medlib.med.utah.edu/WebPath/RENAHTMURENAUDX.html
South African Renal Society	http://www.sa-renalsociety.org/
South African Renal Society	http://www.ssn-sa.com/
Spanish Society of Nephrology	www.sin.it/defaulte.htm
Swiss Medical Societies	http://www.nephro.ch/Nephro/en/medecins/adresses.html
The Hong Kong Society of Nephrology	http://www.hksn.org/
The Renal Association	http://www.renal.org/
The Renal Physicians Association (RPA)	http://www.renalmd.org/
Turkish Society of Nephrology	http://www.tsn.org.tr/default_en.asp
UK Renal Association	www.umds.ac.uk/renal/renalassoc/
Addenbrooke’s Hospital Cambridge Transplant Unit (UK)	http://cambridge-transplant.org.uk/
Bert Fish Medical Center Kidney Transplant Program	http://www.bertfish.com/kidney_transplant_program.html
Karnataka Nephrology & Transplant Institute	http://www.kanti.com/
National Kidney and Transplant Institute of the Philippines	http://members.tripod.com/nktiuro/
Sindh Institute of Urology & Transplantation (Pakistan)	http://www.siut.org/
Washington Regional Transplant Consortium	http://www.beadonor.org/

An e-book is an electronic or digital version of a book. This version of book is predicted to be the future version of books as it is easier to read, requires less space and is easy to browse through and jump to relevant paragraph immediately. Internet has already become a major platform for assessing these books. Few sites allow free browsing and reading of books. While most are restricted to paid members only, some only provide CD-ROM of the book. Table 4 enlists few website of e-books related to nephrology and medicine. Training of nephrologists in advance centers would give them great advantage to explore and learn new technologies; therefore, we have also listed different agencies providing fellowships for advance training for nephrologists [Table 5]. For Indian nephrologists and nephrology-related patients, Table 6 gives useful information on nephrology-related websites of universities, medical centers and government agencies in India.

**Table 4:** Different e-books related to nephrology

Nephrology books	Web address
diesel-ebooks.com	http://www.diesel-ebooks.com/cgi-bin/category/MED055000/Nephrology.html
ebooks.com	http://www.ebooks.com/subjects/b/1193/1.asp
E-books about medicine	http://www.ebooksabouteverything.com/ebooks/category/MED055000/Nephrology.html
Books from Stanford university	http://lane.stanford.edu/online/ebsubjectbrowse.html?m=Nephrology
Louis Calder Memorial Library	http://calder.med.miami.edu/catalog/subject/nephrology.html
FSU College of Medicine	http://med.fsu.edu/library/Ebooks/ebooks.aspx
MediFocus Guidebook	http://www.renalcellcarcinoma-info.com/renal-cell-carcinoma-guidebook.php
FreeBooks4Doctors	www.freebooks4doctors.com
eMedicine	www.emedicine.com
UpToDate	www.uptodate.com
Harrison’s Online	www.harrisons online.com
The online books page	http://onlinebooks.library.upenn.edu/
Bookshelf	http://www.ncbi.nlm.nih.gov/entrez/query.fcgi?db=Books

**Table 5:** List of different agencies providing fellowship for advance training for nephrologists

Agency	Web address
Henry Fort Health system	http://www.henryford.com/body.cfm?id=44841
Mayo Clinic Program	http://www.mayo.edu/msgme/nephrology-rch.html
Georgetown University Hospital	http://medicine.georgetown.edu/nephrology/fellowship_program.htm
Baystate Medical Centre	http://academics.bhs.org/Fellowships/Nephrology_Fellowship.html
Australia and New Zealand Society of Nephrology	http://www.nephrology.edu.au/awardsfellowshipsandgrants/fellowships.asp
Kaiser Permanente Los Angeles Medical Center (LAMC)	http://residency.kp.org/scal/fellowship/nephrology/index.html
University of North Carolina	http://pediatrics.med.unc.edu/div/nephro/fellowship_overview.htm
Allegheny General Hospital	http://www.wpahs.org/agh/education/graduate/nephr/index.html
Nephrology fellowship at VCU	http://www.intmed.vcu.edu/home/fellowships/nephrology.html
University of Alabama	http://www.nrtc.uab.edu/fellowships.htm
Maine Medical Center	http://www.wmhcc.org/mmc_body.cfm?id=1633

**Table 6:** List of useful nephrology-related websites of universities, medical centers and government agencies in India

Name and address of medical colleges/medical institutions	Web address
Osmania Medical College, Hyderabad, Andhra Pradesh	http://www.osmania.ac.in/
All India Institute of Medical Sciences, New Delhi	http://www.aiims.edu/
BJ Medical College, Ahmedabad, Gujarat	http://gujhealth.gov.in/medi_edu/ins_bj_ahm.htm
Seth GS Medical College, Mumbai, Maharashtra	http://www.kem.edu/
Bombay Hospital Institute of Medical Sciences, Maharashtra	http://www.bombayhospital.com/index1.html
Postgraduate Institute of Medical Education and Research, Chandigarh, Punjab	http://pgimer.nic.in/
SMS Medical College, Jaipur, Rajasthan	http://medicaleducation.rajasthan.gov.in/jaipur/index.asp
Christian Medical College, Bagayam, Vellore, Tamilnadu	http://www.cmch-vellore.edu/
Institute of Medical Sciences, Varanasi, Uttar Pradesh	http://www.indiauniversity.me/banaras-hindu-university-varanasi-uttar-pradesh/
Sanjay Gandhi PG Institute of Medical Sciences, Lucknow, Uttar Pradesh	http://www.sgpgi.ac.in/
Sri Venkateswara Institute of Medical Sciences, Tirupati Govt.	http://tirumala.org/activities_social_svims.htm
Gandhi Medical College, Hyderabad	http://www.gandhimedicalcollegehyderabad.org/
Kerala Medical College, Thiruvananthapuram Govt.	http://www.govtmedicalcollegetvm.net/
Sri Ramachandra Medical College and Research Institute Trust, Tamil Nadu	http://www.sriramachandra.edu.in/

## A. Nephrology Journals and Article Search

### PubMed

(http://www.ncbi.nlm.nih.gov/entrez/query.fcgi?DB=pubmed): It is a free web-based literature database of National Library of Medicine and includes over 16 million citations from MEDLINE and other life science journals for biomedical articles back to the 1950s. It is free and the most highly accessed database.

### Scopus^™^

(http://www.scopus.com): Scopus provides broad coverage of the sciences and social sciences, indexing 15,000 journals of chemistry, biology, environmental science, mathematics, physics, engineering, health and life sciences, social sciences, psychology and economics. Scopus also provides citation analysis.

### EMBASE

(http://info.embase.com/embase_com/about/index.shtml): The Excerpta Medica database (EMBASE) maintained by Elsevier Science, is a major biomedical and pharmaceutical database indexing over 7000 international journals in the fields of drug research, pharmacology, pharmaceutics, toxicology, clinical and experimental human medicine, health policy and management, public health, occupational health, environmental health, drug dependence and abuse, psychiatry, forensic medicine and biomedical engineering/instrumentation.

### DARE

(http://www.york.ac.uk/inst/crd/crddatabases.htm): Database of Abstracts of Reviews of Effectiveness (DARE), maintained by the NHS Center for Reviews and Dissemination at the University of York, makes it available for searching via its website. DARE is a database of high-quality systematic reviews of the effectiveness of health care interventions.

### DynaMed

(http://www.dynamicmedical.com/what.php): DynaMed is a clinical reference tool created by a physician for physicians and other health care professionals for use primarily at the “point-of-care.”

### HSTAT

(http://www.ncbi.nlm.nih.gov/books/bv.fcgi?rid=hstat): The Health Services Technology/Assessment Texts (HSTAT) is a free, web-based resource of full-text documents that provide health information and support health care decision making.

There are many sites (e.g., Medical Matrix, Yahoo Health, HealthFinder) that catalog health-oriented sites on the Web which meet certain quality criteria. Another example is CliniWeb, a filtered site (http://www.ohsu.edu/cliniweb/), which has additional unique features based on its goals of providing access to quality-filtered, clinically oriented information.[9]

## B. Chronic Kidney Disease and Web

The WWW is now considered a key source of health information; but the quality and utility of this information has been challenged. Calderón *et al*.[10] have reported on the structural, content, and linguistic barriers for the access of the chronic kidney disease (CKD) information and discuss the implications of limited Internet access to communicating health. Technical (number of hyperlinks), content (number of six core CKD and risk factor information domains included) and linguistic barriers were assessed for websites offered by 12 kidney disease associations.

## C. Renal Transplantation and Web Resource

The last decade has seen an exponential rise in the amount and the nature of information that is available on the Internet about renal transplantation. We have described useful web links for detailed information related to kidney transplant [Table 7] and provided links of important agencies which provide financial support to the nephrology patients, especially dialysis and kidney transplant patients [Table 8].

**Table 7:** List of important website addresses related to kidney transplant

Kidney Transplant Guide	http://www.kidneypatientguide.org.uk/site/kid_trans.php
American Association of Kidney Patients	http://www.aakp.org
American Kidney Fund	http://organdonor.gov/faq.html
Coalition on Donation, Donate Life	http://www.patients.unos.org
National Kidney foundation	http://www.kidney.org.uk/
iKidney.com	http://www.trioweb.org
Dialysis Facility Compare	http://www.ktppp.com/index.html
ESRD Network 5, Mid-Atlantic Renal Coalition	http://www.patients.unos.org
Forum of ESRD Networks	http://www.seopf.org
Kidney Transplant Health link	http://www.transweb.org
Legislative Activity	http://www.niddk.nih.gov
Living Donors Online	http://www.kidney.org
Organ Transplant links	http://www.kidtrans.org/kidtrans2.html
National Kidney Foundation - Making Lives Better	http://www.livingdonorsonline.org
NIDDK Kidney Transplantation	http://www.organdonor.gov/legact.htm
Questions About Kidney Transplantation	http://www.healthlinkusa.com/531B.asp
British Colombia transplant Society	http://www.transplant.bc.ca/research_main.htm
South Eastern Organ Procurement Foundation	http://www.esrdnetworks.org
Transplant Patient Data source Transplant Center Report	http://www.esrdnet5.org
Transplant Patient Partnering Program	http://www.medicare.gov/Dialysis/Home.asp
Transplant Recipients International Organization, Inc.	http://www.ikidney.com
United Network for Organ Sharing Transplantation Resource	http://www.shareyourlife.org
US Department of Health and Human Services	http://www.akfi nc.org
Washington Regional Transplant Consortium	http://www.wrtc.org
Multi-Organ Transplant Program	http://www.lhsc.on.ca/transplant/
American Society of Transplant Surgeons	http://www.asts.org
HDCN Transplant	http://www.hdcn.com/ch/trans
ESRD Network 5, Mid-Atlantic Renal Coalition	http://www.esrdnet5.org
Forum of ESRD Networks	http://www.esrdnetworks.org
Kidney Web	http://www.pw1.netcom.com/~knight10/kidneyweb.html
National Academy of Sciences, Institute of Medicine	http://www4.nas.edu/iom/iomhome.nsf
United Network for Organ Sharing Transplantation Resource	http://www.unos.org
University Renal Research and Education	http://www.urrea.org/about.html
Scientific Registry of Transplant Recipients	http://www.ustansplant.org
United States Renal Data System	http://www.usrds.org
US Department of Health and Human Services	http://www.os.dhhs.gov
National Kidney Research Fund	http://www.nkrf.org.uk/
Transplant Information Website	http://body.orpheusweb.co.uk/lnks.html
Renal-Net	http://www.renalnet.org/renalnet/renalnet.cfm
International Peritoneal Dialysis Society	http://www.ispd.org
American Kidney Fund	www.kidneyfund.org
Transplant Pathology	http://tpis.upmc.edu/

**Table 8:** Different funding agencies helping nephrology patients

Agencies that donate for dialysis and renal transplant	Web address
Universal Giving	http://www.universalgiving.org/
American Kidney Fund, Inc	www.kidneyfund.org
Saskatchewan Health	http://www.gov.sk.ca/
BC Renal Agency	http://www.bcrenalagency.ca/KidneyCare/
American Association of Kidney Patients (AAKP)	http://www.aakp.org/
National Kidney Foundation	http://www.kidney.org/news/newsroom/fsitem.cfm?id=30
Transplant Australia	http://www.transplant.org.au/
SouthWest Transplant Alliance	http://www.organ.org/
The Kidney Transplant/Dialysis Association, Inc.	http://users.rcn.com/ktda1/
New England Organ Bank	http://www.neob.org/
United Network for Organ Sharing (UNOS)	http://www.unos.org/
Organ Procurement and Transplantation Network (OPTN)	www.kotb.com
The British Kidney Patient Association	http://www.britishkidney-pa.co.uk/
National Kidney Foundation of Connecticut	http://www.ctorganandtissuedonation.org/memmber_agencies.html

## D. Useful Nephrology Links

### Nephrology@MedNet

The largest collection of direct links to online medical search engines and databases. Find the Web’s free and extensive medical archives, libraries, research databases, catalogs, and statistical data available at educational, governmental and corporate sites.

### CyberNephrology

E-mail discussion group for parents of children with kidney disease. cyberNephrology^™^ provides this website for the purpose of rendering online services, providing useful information and spreading knowledge (http://www.cybernephrology.org/).

### iKidney.com

Nephrology pediatric educational material but no chat room. iKidney has been selected as a featured site in Lightspan’s StudyWeb^®^ as one of the best educational resources on the web. StudyWeb^®^ is an educational resource site for students and teachers that select only the important informative websites (http://www.ikidney.com/).

### Kidney Patient Guide

For kidney patients and those who care for them (http://www.kidneypatientguide.org.uk/site/contents.php).

### CROWNWeb

The Centers for Medicare and Medicaid Services is developing a web-based application, Consolidated Renal Operations in a Web-Enabled Network (CROWNWeb), which is designed to facilitate data entry, updating, and retrieval for dialysis facilities nationwide. Part 1 of this three-part series outlines the history of end-stage renal disease and Medicare, and covers the rapid growth of ESRD (End stage renal disease) data management as well as other events that require progression to an online data collection and management system.[11]

### HONcode

Health on the Internet Foundation Code of Conduct (HONcode) for medical and health websites (http://www.hon.ch/HONcode/index/html) specifies eight principles to direct website developers in basic ethical standards and discover that consumers know the source and rationale of the data they are reading. To assist consumers use and comprehend medical terminology, the Medical Library Association (MLA) has published a brochure entitled ’’Deciphering Medspeak’’ which is offered free of charge in individual copies by sending an e-mail to info@mlahq.org or visiting the MLA website at http://www.mlanet.org and for bulk orders the e-mail has to be sent to mlafa@mlahq.org The MLA has provided a “top ten” list of most useful consumer health websites.[12]

## Database of Individual Patients’ Experience

Database of Individual Patients’ Experience (DIPEx) is a new Internet-based multimedia resource that provides access to the experiences of others who have faced similar health dilemmas. On the DIPEx site, anybody can watch video clips, listen to the voices or read the accounts of people relating their experiences of illness and the impact it had on their lives. We can also find out about an illness and its available treatments, and find links to support groups and other reliable sources of medical information. DIPEx aims to promote more balanced encounters between patients and health care professionals by providing detailed access to the patient perspective. It is, at once, a 24-h support group for anyone whose life is touched by an illness and a valuable resource for the training of doctors, nurses and other health professionals and health workers.

Home dialysis center (http://www.homedialysis.org/resources/stories/) is a very useful site for nephrology patients to obtain important and critical information regarding their future care.[13]

## Patient Stories and Experiences and Their Impact on Other Patients and Health Professionals

Internet (e-patients.net) is a very powerful tool for search and retrieves information from stories of other patients. With a “story topics search” facility, participants are better able to find the information they are looking for. Also, they retrieve stories that more closely covered, their information needs and they learn more from the stories retrieved.[14]

## Challenges and Limitations

Reliability, discretion and security are the major concerns for application of Internet in nephrology. Various antiviruses like Norton-Antivirus (www.symantec.com), f-prot (www.f-prot.com), Thunder-BYTE (http://www.thunderbyte.com/), McAfee (http://www.mcafee.com) are available, which can be effectively utilized not only for repairing infected programs but also checking virus entry and enhancing cyber security. With a rise in numerous of health care websites, clinicians and patients have more information than ever to wade through, even simply the volumes of information it contains can be exhausting to search out for specific need. Unfortunately, many of these sites contain incomplete, misleading, or difficult to understand information, while others blur the distinction between advertising, medical advice and simple disclosure of fact.[15]

At present, spurious health care websites have outnumbered the trustworthy ones, commonly that of universities, medical centers, government agencies, etc. Some patients consider online consultations as substitutes for a physician’s visit, ignoring the disclaimers that the Internet information is not a substitute of medical practices. Therefore, in order to prevent the spreading of wrong information as well as making the user aware of these sites, the management of quality and reliability of medical information on Internet is extremely important. A possible solution could be labeling of medical information websites by web authors. Labeling and filtering technologies such as platform for Internet content selection (PICS) can help separate valuable health information from doubtful information. Further, doctors, medical societies and related people can critically evaluate Internet information by putting electronic evaluative and descriptive “tags” on it. Table 9 provides more insight to check the authenticity of information and focuses upon health misinformation, fraud and quackery as public health problems.

**Table 9:** Important websites associated in relation to scientific facts and quality management of medical information

Websites	Related information
http://www.acsh.org	separate scientific facts from nonsense
http://g.webring.com/hub?ring=antiquackerysite	Combat and debunk health-related frauds, myths, fads, and fallacies, and are more interested in real, objective, scientific proof, than in the speculative, subjective, and unproven theories and anecdotes of so-called Alternative Medicine
http://www.bmj.com/cgi/content/full/317/7171/1496#art	Quality of information on Internet
http://www.dietfraud.com	Inform public about fraudulent diet products
http://www.chirobase.org	A skeptical guide to chiropractic history, theories and current practices. Accurate information about chiropractic is not easy to get. This Website enables to deepen understanding. If you decide to seek chiropractic care, it may also help find a suitable practitioner
http://www.ftc.gov/bcp/consumer.shtm	Practical information on a variety of consumer topics
http://www.ncahf.org	NCAHF is a private nonprofit, voluntary health agency that focuses upon health misinformation, fraud and quackery as public health problems
http://www.fraud.org/	National Consumers League tracks complaints of inappropriate health care practices works in conjunction with the Federal Trade Commission, National Association of Attorneys General
